# Supplementary material for: Increased Expression of Cell Surface SSEA-1 is Closely Associated with Naïve-Like Conversion from Human Deciduous Teeth Dental Pulp Cells-Derived iPS Cells
Source: Int J Mol Sci. 2019 Apr 3;20(7):1651. doi: 10.3390/ijms20071651 (PMC6480091; doi:10.3390/ijms20071651)
Supplement: Supplementary file 1 [file ijms-20-01651-s001.pdf]

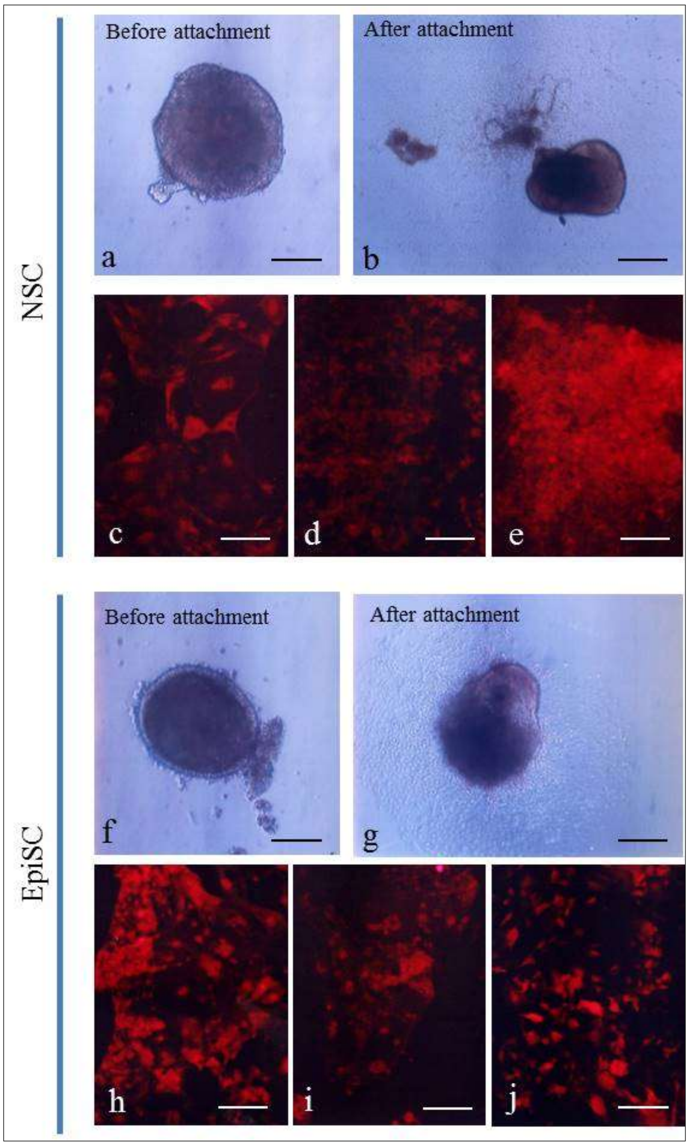

Suppl. Figure 1. In vitro differentiation induction of HDDPC-derived iPSCs. NSC-like colonies (NSC) generated 4 days in NSC culture after the 4th passage and EpiSC colonies (EpiSC) were induced to form EBs, as described in Materials and Methods, and then the resultant EBs were placed in the tissue-culture dish to allow attachment on the dish and subsequently form an outgrowth of differentiated cells. After 10 days of attachment of EBs to the substratum, the presence of differentiated cells from three germ layers (endoderm, mesoderm, and ectoderm) were confirmed by immunocytochemical staining of differentiated cells using typical endodermal (AFP; for c,h), mesodermal (α-SMA; for d,i), and ectodermal (βIII-tubulin; for e,j) markers. Bar = 100 μm.

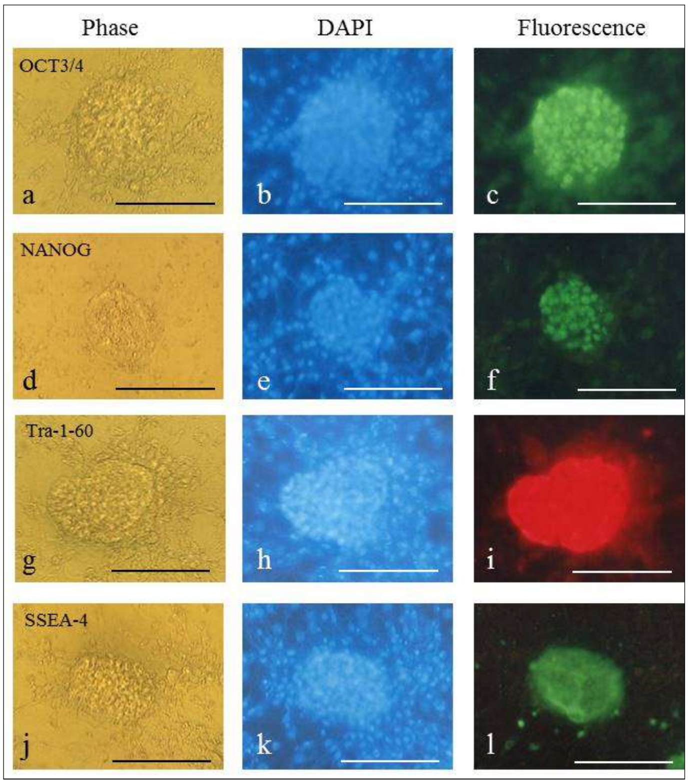

Suppl. Figure 2. Immunostaining of NSC-like colonies (generated 4 days in cultivation with NSC medium after the 4th passage) using various antibodies raised against OCT3/4, NANOG, Tra-1-60 and SSEA-4. Phase, photos taken under light; DAPI, photos taken under UV illumination for detection of the DAPI-derived fluorescence; Fluorescence, photos taken under UV illumination for detection of the second antibody-derived fluorescence. Bar = 200 μm.

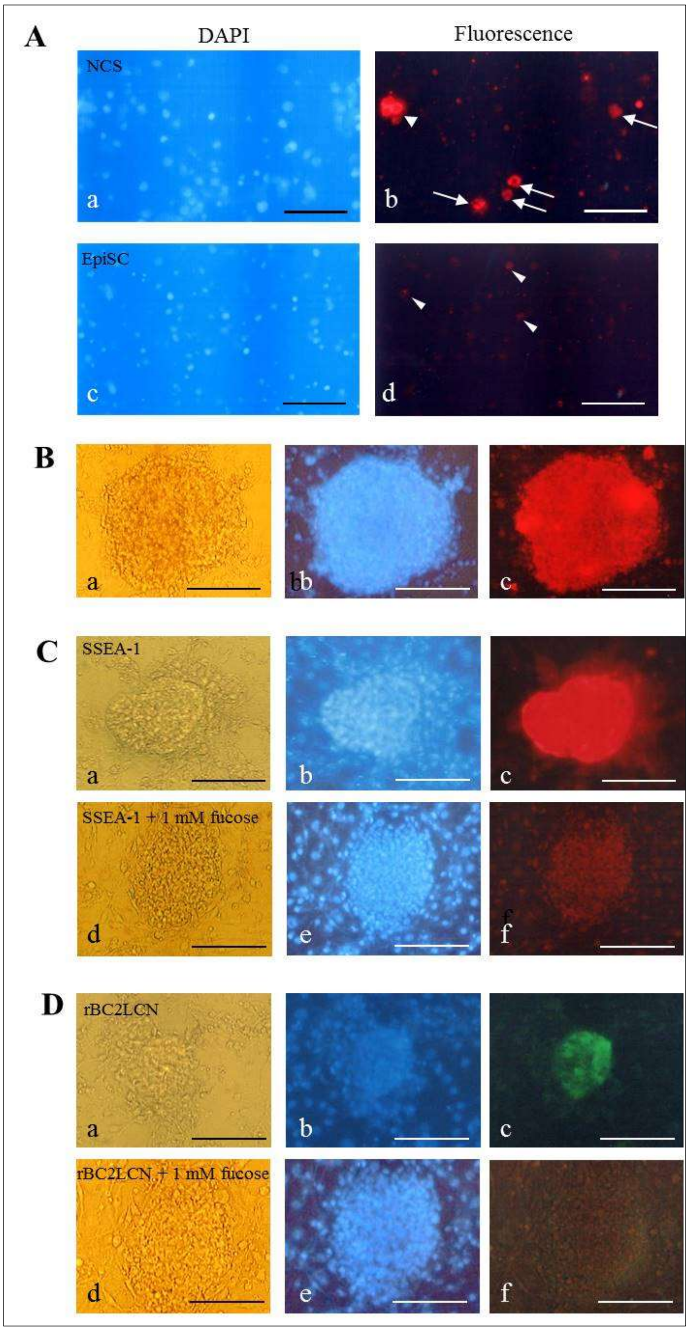

Suppl. Figure 3. Staining of HDDPC-derived iPSCs using anti-SSEA-1 and rBC2LCN lectin. **A.** Immunostaining of partially dissociated NSC-like colonies (**a**,**b**) or EpiSC colonies (**c**,**d**) by anti-SSEA-1 (Lot no. GR38347-10). DAPI, photos taken under UV illumination for detection of the DAPI-derived fluorescence; Fluorescence, photos taken under UV illumination for detection of the secondary antibody-derived fluorescence. Bar = 200 μm. **B.** Immunostaining of NSC-like colonies with another lot (Lot no. GR287616-11) of anti-SSEA-1. **a**, photo taken under light; **b**, photo taken under UV illumination for detecting DAPI-derived fluorescence; **c**, photo taken under UV illumination for detecting the secondary antibody-derived fluorescence. Bar = 200 μm. **C.** Immunostaining of NSC-like colonies with anti-SSEA-1 (Lot no. GR38347-10) (**a-c**) or with anti-SSEA-1 + 1 mM fucose (**d-f**). **a**,**d**, photos taken under light; **b**,**e**, photos taken under UV illumination for detecting DAPI-derived fluorescence; **c**,**f**, photos taken under UV illumination for detecting the secondary antibody-derived fluorescence. Bar = 200 μm. **D.** Cytochemical staining of NSC-like colonies with FITC-labeled rBC2LCN (**a-c**) or with FITC-labeled rBC2LCN + 1 mM fucose (**d-f**). **a**,**d**, photos taken under light; **b**,**e**, photos taken under UV illumination for detecting DAPI-derived fluorescence; **c**,**f**, photos taken under UV illumination for detecting the rBC2LCN-derived fluorescence. Bar = 200 μm.
